# Unreliable conspiracies: survey results about COVID-19 conspiracy theories lack temporal stability

**DOI:** 10.1192/j.eurpsy.2023.1724

**Published:** 2023-07-19

**Authors:** V. Pisl, J. Volavka, J. Vevera

**Affiliations:** Psychiatry clinic, Faculty of Medicine in Pilsen, Charles University, Plzen, Czech Republic

## Abstract

**Introduction:**

Conspiracy theories complicating public reaction to the COVID-19 pandemic inspired quantitative research on conspiracy theories, mostly using survey-based, correlational designs. Data from similar studies may, however, be unreliable due to low temporal stability (Graham, 2021).

**Objectives:**

We examine the temporal stability of a popular survey measure of COVID-19 conspiracy beliefs (CCBs).

**Methods:**

CCBs were measured by a popular set of items developed in the first months of the pandemic, addressing the beliefs that COVID-19 was a hoax (CCH) and that it was artificially created for evil purposes (CCC) (Imhoff & Lamberty, 2020), in 179 students of medicine. In March 2022, CCBs were measured twice using the same set of questions presented once with a numeric (N1 measure) and once with a Lickertian (L1 measure) scale, with filler questions in between. The same Lickertian items were presented to the same sample in May 2022 (L2 measure).

**Results:**

The mean agreement with CCBs did not differ between March and May 2022 and previous survey on a similar sample in January 2021. The temporal stability of CCBs expressed as the correlation between the L1 and L2 measurement was poor (r = .57 for CCC, r = .67, for CCH). The difference between L1 and L2 was positively correlated with agreement with CCBs (r =.21, p < .01 for CCH; r = .44; p < .001 for CCC). Out of 18 respondents reporting agreement with CCC in March and 5 respondents reporting agreement with CCH, only 8 still reported agreement with CCC and 1 reported agreement with CCH in May.

Finally, participants were split based on their L1 CCC score into groups of “mainstreamers”, “undecided”, and “conspirators”. For “mainstreamers”, there was no difference between their CCC score recorded in N1, L1, and L2. For “undecided”, there was a difference between L1 versus N1=L2, suggesting random effects (regression to the mean). For “conspirators”, the scores recorded in March were equal, while their agreement with CCC was lower in May, suggesting that the scores recorded in March were not random and the difference between March and May is better explained by situational factors.

**Conclusions:**

Temporal stability of survey-reported CCBs is low, particularly among those reporting agreement with CCBs: When a respondent reports agreement with a CCB in a survey, they are more likely to disagree than agree with the same CCB two months later. The low temporal stability seems to be affected not only by incorrect or random answers, but also by situational factors. Implications: First, survey measures of CCBs may inflate the spread of conspiracy theories in population. Second, correlations of CCBs with other variables measured by surveys may be inflated via *common method bias*, distorting our understanding of the predictors and consequences of CCB.

**Disclosure of Interest:**

None Declared

